# Facilitating collaborative professional development among instrumental and vocal teachers: A qualitative study with an Austrian Music School

**DOI:** 10.3389/fpsyg.2022.1096188

**Published:** 2023-08-11

**Authors:** Silke Kruse-Weber, Elizabeth Bucura, Margareth Tumler

**Affiliations:** Department of Music Pedagogy, University of Music and Performing Arts Graz, Graz, Austria

**Keywords:** instrumental music teachers, collaborative reflection, professional development, empowerment, professionalization, facilitation, teaching and learning enhancement, music school

## Abstract

This case study provides an in-depth investigation in a professional development project about facilitating collaborative reflection. This was led by a research team from the university with 13 instrumental music teachers from one music school in Styria (Austria) during 2019–2021 (including the initial COVID-19 pandemic). Research questions considered (1) the participants’ descriptions of the collaborative professional development, (2) participants’ uses of reflection tools and indications of their identification with workshop interventions, as well as factors responsible for the outcomes from the reflection tools; and (3) ways participants’ thinking and attitudes may have developed through the workshops, how they defined themselves as a group (if they did), and how they might have gained trust in one another. Inspired by the design-based research approach, practitioners and researchers worked closely together to enhance teaching and learning implementing interventions with collaborative reflections tools. While the first phase (11 workshops) was primarily led by the project-team, the second phase (7 workshops) was participant-led. Data included focus groups and discussion transcriptions from 18 workshops. The impetus of the study included the role of the director and the participants dealing with the interventions, and finally the participants’ descriptions of their experiences in the professionalization process. Literature included collaborative professional development, community of practice, learning communities, self-determined learning, reflective practice, and ethical considerations. Data were analyzed based on thematic analysis and gave rise to five following themes: forming group cohesion, inspiring and appreciating collaboration, bridging theory and practice, identifying deeper thinking and teachers as learners, addressing challenges and potentials during the COVID-19 pandemic, and finally finding the music school’s own identity and sense of importance. Findings highlight the importance of establishing meaningful collaborative reflection through appreciative communication and an atmosphere of trust and respect. To be able to make change in and with an institution, leadership members must be engaged as collaborative stakeholders on an eye-level; collaborative professional development can be used as a resource toward rethinking and reworking the identity of one’s music school and of teaching and learning. Institutions should provide space and continuity for such development. Finally, the study highlights that a collaborative reflective approach can contribute to professional and social growth.

## 1. Introduction

This qualitative case study investigated 13 instrumental and vocal music teachers from a public music school in Styria, who collaborated with a research team in collaborative reflection. First, we provide the background and theoretical framework of the study. Second, we outline the design of the workshops. In the method section we describe our analytic approach before presenting and discussing results. Finally, we suggest professional development projects how to focus on collaborative professionalization.

The rationale and background of this study reference a longstanding problem in music pedagogy. The terms theory and practice are often used dichotomously, reflecting a complex tension (Vogt, 2002; Lehmann-Wermser and Niessen, 2004; Niessen and Richter, 2011; Kruse-Weber, 2018). Newer paradigm shifts that consider a broad view of societal and political issues only slowly take root, hindering development and professionalization (Kruse-Weber, 2018). Gaunt (2016) and Westerlund et al. (2019) point out that society, music-making, and educational institutions undergo dynamic changes, while instrumental and vocal teaching often continue along traditional paths. Bransford et al. (2005) noted these traditions affect teaching decisions and should be examined. Therefore, in instrumental and vocal teaching, there is increasing need to develop reflective and lifelong learning to respond to social changes in flexible and collaborative ways (Shuler, 1995; Smilde, 2009; Westerlund et al., 2019). Relatively few studies—specifically in the German-speaking context—have systematically examined professional development activities from experienced instrumental/vocal teachers (Bauer, 2007; Conway, 2007; Conway, 2008; Reynolds et al., 2010). Brewer and Rickels (2014) provide an overview of extant research on the professional development of music educators from Hookey (2002) and the Journal of Music Teacher Education, which devoted a special issue to the topic (Conway, 2007). Mostly studies on professional growth focused on how technology and the Internet can be used to improve music-teaching practices. The study from Brewer and Rickels (2014) about professional growth opportunities of music educators within an online social media community are aligned with communities of practice that exhibit common characteristics. New studies, such as Westerlund et al. (2019), Gaunt and Westerlund (2022), Westerlund (2020), and Westerlund et al. (2021) about professionalism in music (education) argue that music schools as social-ecological spaces ought to develop *institutional resilience* (Senge, 2006; Westerlund et al., 2019). The resilience of music schools as social-ecological systems is important toward renewal, re-organization and development of social structures to generate new values and enhance sustainability through innovations (Westerlund et al., 2019). Clear connections should exist between initial professionalization in terms of teacher education, and continued development for in-service teachers. Therefore, our study, while focused on professional development, may also have implications for teacher education.

In this study, we consider that professionalization involves a process of defining group norms and purpose, not only day-to-day decisions, but also a greater sense of cultural and societal purpose, as well as ethics by which to guide one’s practices. Related to Gaunt and Westerlund’s (2022) concept of professionalism, our view comprises music teachers’ senses of agency, position in relation to a wider society, and resilience. We consider that through professionalization, one may claim belonging and expertise as a bound group. Society, too, may come to recognize the group as professionals (in recognition of expertise unique to them) and music teachers can come to therefore feel respected and appreciated for their work. This can lead to a sense of confidence, camaraderie, skills, and expertise, furthering satisfaction in one’s work.

Along these lines, and with intentions to bridge a gap between innovations in pedagogical theory and practice, in 2016–2018 we worked with colleagues in the project *Network IGP*^1^ from the University of Music and Performing Arts Graz (KUG), Austria on collaborative professional development (CPD; Kruse-Weber, 2018). In order to expand on the teachers’ experiences of CPD, and also to bring it to music schools, we initiated a follow-up project called *Reflective Practice in Innovative Music Schools.*^2^ We intended to establish a group of teacher-learners, to expand their teaching expertise, and to empower them to take on new perspectives. These include the awareness of understanding teaching and learning in a broader context of social systems and as a constitutive issue of social justice and “politics of memory” (Bowman, 2006; Odendaal and Westerlund, 2022).

**Figure 1:**
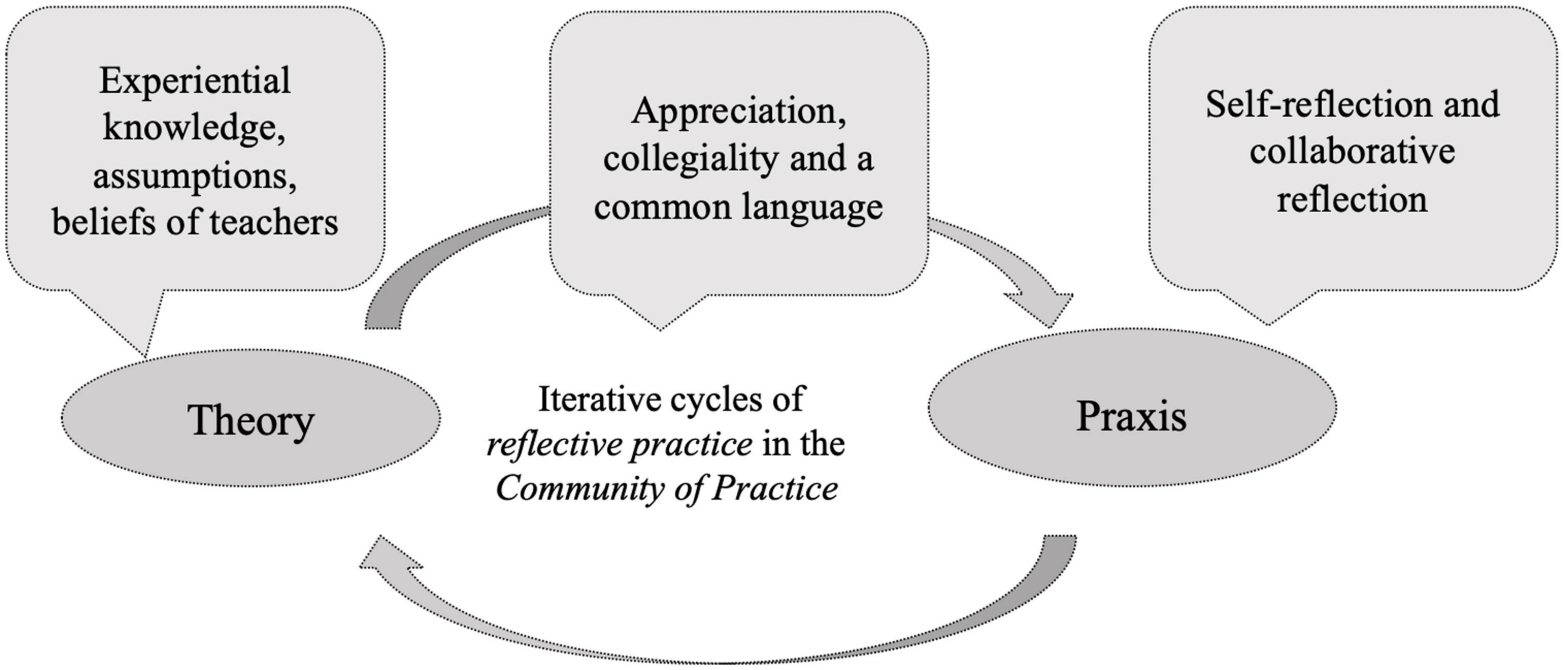
Learning as co-construction in social interaction—reflection principles, bridging theory and practice, adapted from Gaunt and Westerlund (2013) and Kruse-Weber (2018).

Our rationale for this study can be framed as shown in Figure 1.

This CPD project occurred over two periods: The first Phase (*IGP-Go,*^3^ with 11 workshops from January 2020 to January 2021) involved facilitated meetings, use of reflection tools and group discussion. The collaborative reflection of these workshops led to the idea of forming a task force, which developed in a Second Phase (*A Music School Speaks,* with seven work meetings from May to October 2021) and involved a shared vision for innovative and contemporary music school work. This concept was presented at the conference *Challenge Accepted 4.0,*^4^ aimed at music school teachers’ collaborative exchange (Tumler and Kruse-Weber, 2022).

The purpose of this study, was to deepen our understanding of instrumental music teachers (referred herein as instrumental teachers, also implying vocal teaching): of their work, teaching approaches, attitudes, experiences and receptiveness to potential change or growth, and their perspectives on problems, barriers and potentials of knowledge transfer with their students. As authors have noted, collaborative reflection and facilitation is important as an integral part of building group cohesion (Kruse-Weber, 2018; Kruse-Weber and Tumler, 2020; Tumler and Kruse-Weber, 2022).

Before proceeding, some terms require explanation. In this study, we discuss professional development as a continuation of professionalization, indicating further growth in articulating one’s purpose and goals, gaining support, and refining one’s practice. Drawing from the work of other researchers (e.g., Hakkarainen et al., 2004; Hakkarainen, 2013; Timonen, 2021), we regard professional development as purposeful and curiosity-driven interactions between individuals and groups, in order to develop new knowledge and learning. As Darling-Hammond et al. (2005) noted, CPD is necessary in that teaching itself is a complex act in which one must integrate different knowledge and skills in order to make decisions about how to achieve goals with diverse learners. Reflection processes (both collaborative and self-reflection) played important roles in this study. We regarded reflection as on-action, rather than in-action, as participants viewed their own teaching recordings and considered them after some time and within a new context (Schön, 1983). Deep learning outcomes, as discussed by Marton and Saljo (1976), can be defined as those that collectively comprise understandings, and one’s ability to apply those understanding (as opposed to simply memorizing or imitating information). Lifelong learning involves curiosity and motivation to seek growth in one’s life, including professional life. Bucura (2020b) noted the importance of continued learning for music teachers in terms of support, balance, and sustainability. Music teachers’ professional knowledge is related to professionalization explained earlier, including defining group norms and a sense of purpose and ethics. In this study, self-determined learning involves an application of learning in practical contexts, which necessitates that the learner (or in this case teacher-learner) is flexible, resourceful, and can make needed adaptations, even when transferring to varied and changing contexts (Bucura, 2020a). Collaborative professional learning included delineating tasks that collectively seek a common aim, defining roles in relationship to one another, and reflecting on these processes toward cohesion as community and personal and group growth.

**Figure 2:**
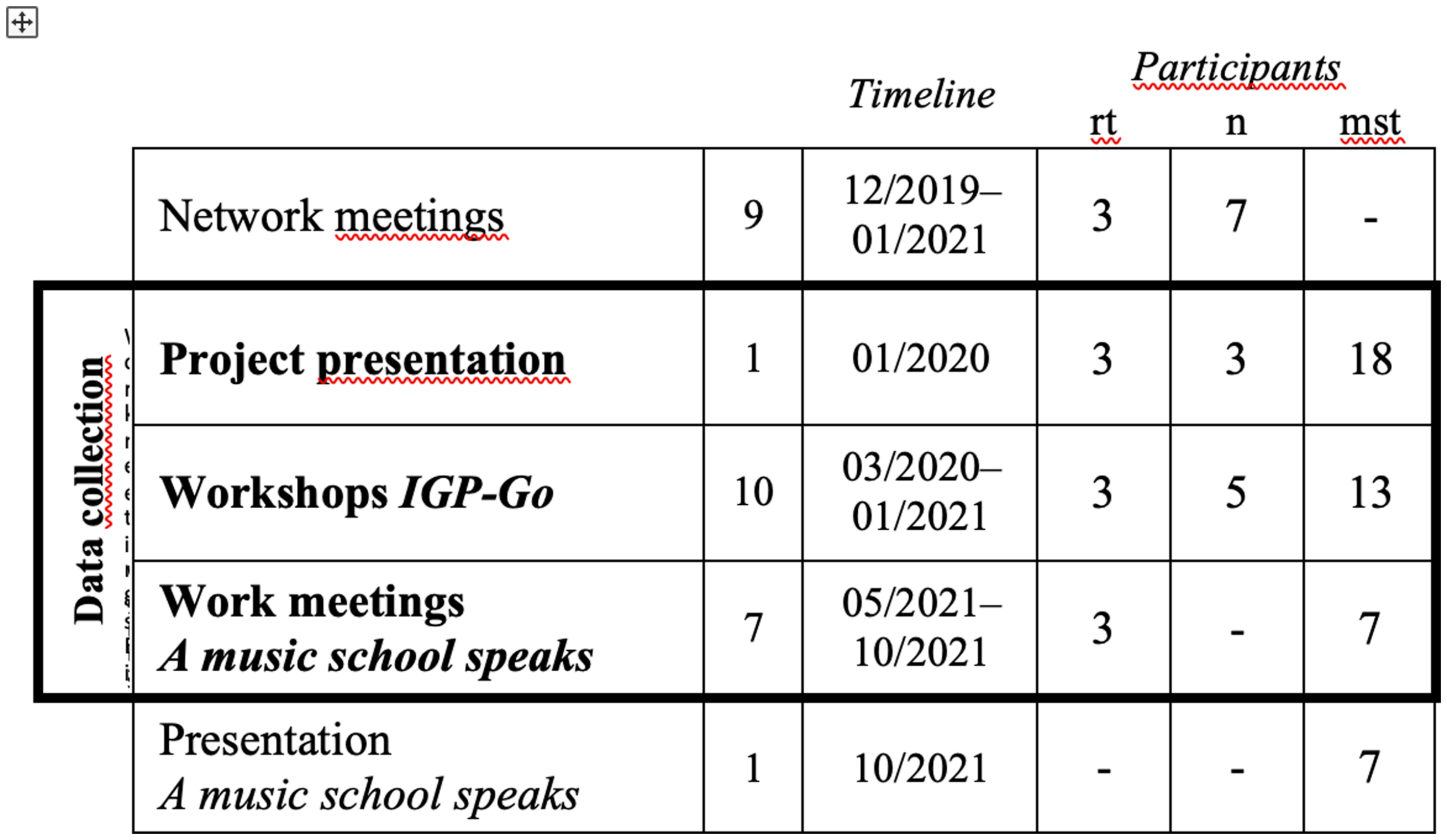
Overview of the project *Reflective practice in innovative music schools*. The CPD then evolved five colleagues from the university (*networkers*) who had participated in the previous project 2016–2018.

Online learning and collaboration were not originally intended as an aspect of this study, and therefore, are not explicitly outlined as a research goal. Yet, due to the unanticipated outbreak of the COVID-19 pandemic, six of the 18 workshops were held online and hybrid. Changes necessitated by the pandemic emerged as an important aspect of participants’ (and our own) experiences. Which gives information about the participants in the phases. For an overview of the project phases, see Figure 2.

Following research questions were considered:

1. How can the professional development process of the participants be described?
2. How did participants collaboratively discuss using the reflection tools and how did they indicate their identification with workshop interventions? What factors supported the outcomes from the reflection tools? Which problems, barriers, and potential benefits of the interventions did participants describe?
3. How and why might participants’ thinking and attitudes have developed through the workshops? How did they define themselves as a group (if they did)? How did participants describe viewing themselves professionally?

In the following we unfold a theoretical framework for the study. Participation in the workshops changed over time. Workshops began with time to visit socially. Enhanced perhaps by significant time (length of workshops and amount of workshops over time), we felt participants formed what became a community of practice (CoP; Wenger, 1999). They reflected on the interventions that included talks from the first author and her facilitation of the reflection tools. We define intervention as the implementation of innovative (reflection) tools for learning and teaching, which as opposed to interference can be regarded as a positive challenge to one’s thinking in becoming intentionally involved in order to improve it.

The CoP supported a shared interest in teaching practices, allowing them to create new knowledge to advance professional practice personally and affecting the music school. While we later reference overlapping aspects of the group with what could also be considered a professional learning community, goals of improving students’ learning were only tangential to the group’s purpose and rather focused more explicitly on their own growth. Therefore, we regard participants as a community of practice.

Literature involving themes of identity and value specific to music schools and private instrumental (and vocal) teaching are lacking, but some points are noteworthy. In some countries, music teacher education tends to focus squarely on school music teaching, sometimes neglecting practices specific to instrumental and vocal teaching (Bucura, 2013; Bucura, 2022), and sometimes failing to provide meaningful professional development and support for practicing instrumental teachers (Bucura, 2020b). Programs have been criticized for lacking a clear connection among required coursework as well as shared values and conceptions of teaching music among faculty (e.g., Darling-Hammond et al., 2005; Hammerness, 2006a). According to Darling-Hammond et al. (2005), the content of coursework matters less than a focus on *how* one learns. These inconsistencies may contribute to a lack of cohesion and vision among instrumental and vocal teachers in music schools, as well as underdeveloped senses of professional identity among them, perhaps without a unifying vision (Hammerness, 2006a). Some programs may offer only what Darling-Hammond et al. (2005) referred to as disjunct, piecemeal domains of the curriculum.

As Grossman et al. (2009) stated, foundations and methods courses should be aligned, bridging theory and practice, and encouraging cohesion between university preparation programs and music schools, as well as encouraging development of not only knowledge and skills, but also professional identity. Other policy areas are also important in order to support good teachers, which—specifically related to this study—include professional development, mentorship, and feedback, along with others like thoughtful retention, preparation, induction, and career development (Darling-Hammond et al., 2017). These areas may contribute to a teacher’s educational vision, which Hammerness (2006b) notes is lacking in education widely, and when present tends to articulate only institutional hopes rather than to guide decisions in personal ways. In fact, according to Hammerness (2003), teachers’ own visions may help one understand not only what and how one teaches, but their likelihood of staying in the profession.

In this study, collaboration was important. Gaunt and Westerlund (2013) noted the ways in which it might promote innovation and negotiation of cultural differences and meanings, which seemed to be the case in this study. According to Gaunt and Westerlund (2013), collaboration promotes innovation and the ability to negotiate cultural differences and meanings. Creech and Hallam (2017) point out that it is “interdependence, interaction and mutuality that undergird the creative potential of groups” (p. 58). As Niessen et al. (2014) stated, music pedagogy research can actually promote institutional teamwork, broadened perspectives, and challenge individuals to question themselves. The relationship of the research study to the professional development workshops appeared to facilitate such an exchange.

**Figure 3:**
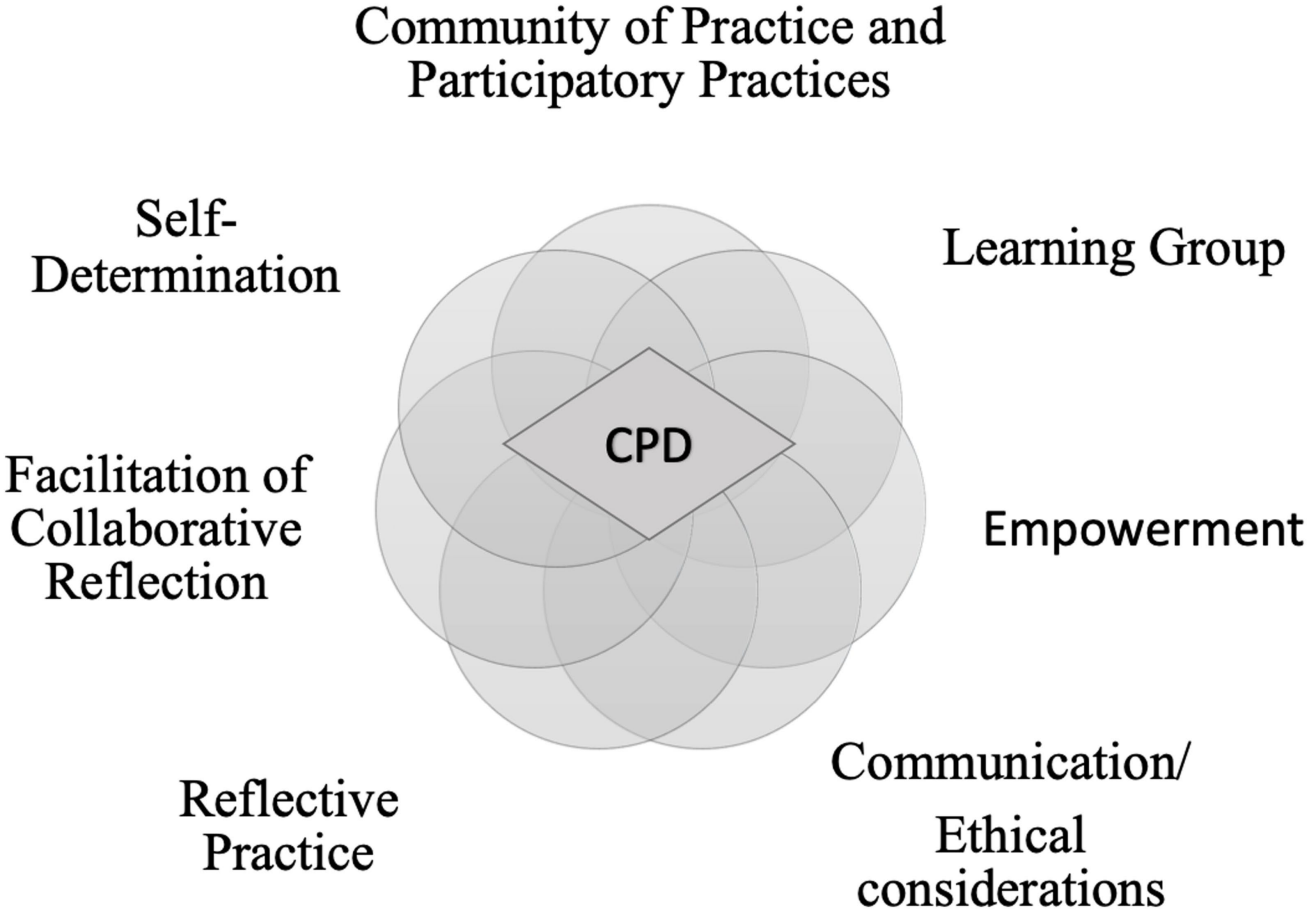
Theoretical framework of the case study *Collaborative Professional development* (CPD).

Collaboration is accompanied by the view that individual learning is regarded as socially situated (Lave and Wenger, 1991). Teachers are thereby regarded as facilitators and co-learners, empowering learners to take ownership of their learning. As Renshaw (2009) describes, “Facilitating is a dynamic, non-directive way of generating a conversation aimed at enabling or empowering a person(s) to take responsibility for their own learning and practice.” (p. 3) In the context of lifelong learning, CPD efforts are increasingly meaningful and successful. In addition to our own experiences from the first project 2016–2018, we ground our project on research-based indicators for effective CPD (see Figure 3), described next.

Stanley et al. (2014) pointed out, one component of CPD consistently identified as meaningful by in-service teachers is collaboration. Since the field of instrumental and vocal education is dynamic, teachers’ careers increasingly become portfolio careers (Polifonia, Erasmus Network for Music, 2010), necessitating such reflection across settings and roles. Feiman-Nemser (2001) stated that

By engaging in professional discourse with like-minded colleagues […] teachers can deepen knowledge of subject matter and curriculum, refine their instructional repertoire, hone their inquiry skills, and become critical colleagues (p. 1042).

Despite value, music teachers in school settings may lack meaningful collaboration, feeling they must simply “sink or swim” without support (Ballantyne, 2007, p. 184). One indicator for effective collaboration in CPD is the ability to empower the teacher-learners not only to expand their expertise but also to empower them to make change. Kirkman and Rosen’s (1999) investigation of work teams in organizations indicated that empowered teams became more productive and proactive than less empowered teams, and had higher levels of job satisfaction and organizational and team commitment. According to them, empowerment comprises (1) self-efficacy of the group with the belief to perform well; (2) meaningfulness, a belief that a group performs important and valuable tasks; (3) autonomy, having independence and discretions in work; and (4) impact, experiencing a sense of significance in the work and goals achieved. Similarly, in CoP, people “share a concern or a passion for something they do and learn how to do it better as they interact regularly” (Wenger, 2006, p. 1). Over time, these individuals develop a shared repertoire of practices “in the form of experiences, stories, tools, and ways of addressing recurring problems” (Miksza and Berg, 2013, p. 7–8).

Highly relevant to the topics of empowerment, CoP and learning groups, are studies of self-determination theory (SDT), which examine the extent to which one meets or frustrates three basic needs: (1) *autonomy*, experiencing interest and values. (2) *Competence*, “feeling of mastery” and that one can succeed and grow, which is best satisfied within “well-structured environments that afford optimal challenges, positive feedback, and opportunities for growth.” (3) *Relatedness* involves a “sense of belonging and connection” (Ryan and Deci, 2020, p. 1).

In addition, reflexivity and reflection are key competences in professional pedagogical action. They can be acquired and provide opportunities to further develop teaching. The attitude of the “reflective practitioner” (Schön, 1983, 1987) ideally describes pedagogical action as an interaction of planning, analysis of situational demand and adaptation to the given teaching situation, and allows reciprocity of theory and practice. Reflective practice allows one to become aware of their strengths through a resource-oriented, reflective attitude (Kruse-Weber and Hadji, 2020). Related, Westerlund (2020) emphasized the importance of reflection through narrative and story in developing senses of community identity, and ways in which narration can lead to transformative professional change.

## 2. Materials and methods

### 2.1. Facilitating the workshops for collaborative reflection

**Figure 4:**
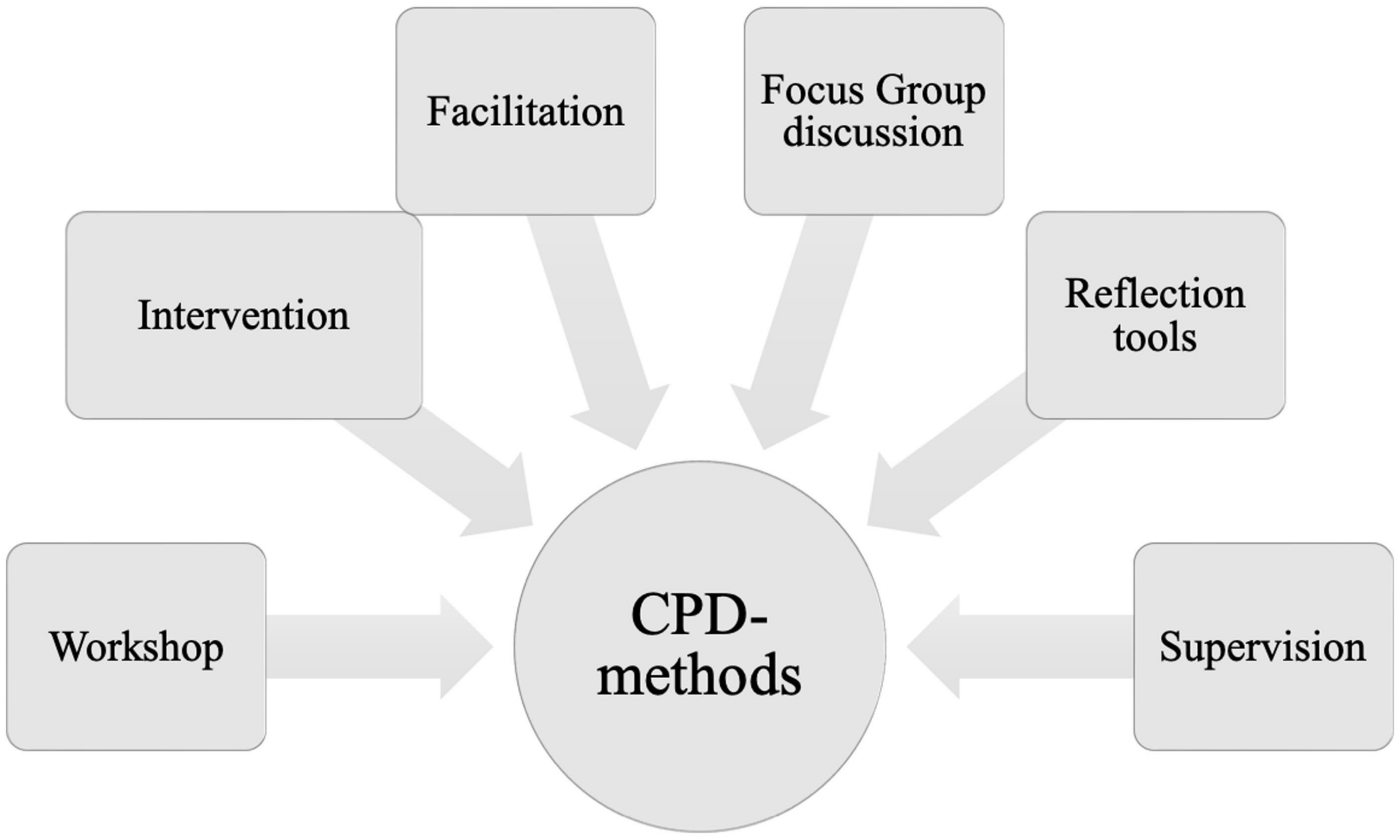
Methods for *Collaborative Professional Development* (CPD).

This case study (Stake, 2000) is bound by a focus on participants’ attitudes, beliefs, behaviors and needs (Williamon et al., 2021). Data were generated from focus groups that provided insights about experiences, attitudes, opinions, expectations, and cultural understandings (Stewart and Shamdasani, 2015; Williamon et al., 2021)—and from the discussion of the participants in the second cycle *A Music School Speaks*. These data contributed to “community building and emancipatory effects among participants” (Bär et al., 2020, p. 215). In the following we clarify methods used to facilitate the project: See Figure 4 for the activities in professional development, we held workshops once per month, working as an intensive group on a practice-oriented topic for approximately 2.5 h.

Focus discussions needed to be carefully planned to obtain participants’ perceptions, including maintaining an open tone. The interaction of respondents could have undesirable effects (a particularly opinionated member could bias results, or conversely, a more reserved group member may be hesitant to talk). We encouraged participants to share their thoughts in an established safe environment. The facilitation by the first investigator was designed to elicit the most compelling and telltale responses from the participants. We knew that the quality of learning and teaching would not be easily captured, depending on participants’ conceptions of good teaching (Carbone et al., 2019). We also were aware about the inefficiency of unfolding an “expert delivery” format, which would set narrow normative frameworks for the participants (Jørgensen, 2009). Rather, we recognized a need to discuss, explore, and reflect quality of teaching and learning mutually. Additionally, reflection was informed by research and educational theory, so our individual experiences could be “related to broader viewpoints and clarifying overviews and theories” (Jørgensen, 2009, p. 111–112).

Inspired by workshops held by the European higher music education initiative *Innovative Conservatoire* (*ICON*), reflection tools were explored with participants in focus groups and further developed in the sense of a community of practice. Based on a number of group characteristics, the group could also be regarded as a professional learning community (Mitchell and Sackney, 2011). Stoll et al. (2006) point out the characteristics of a learning group such as shared values, collective responsibility for pupils’ learning, collaboration focused on learning, group cohesion and individual professional learning, reflective professional enquiry, mutual trust, respect and support. Despite these shared points, however, we regard the participants more specifically as a community of practice in that collective responsibility for pupils’ learning (Mitchell and Sackney, 2011) was only a related aspect rather than direct focus of the group.

The tool *Sources* aimed to share and reflect one’s own understanding of teaching and learning while upholding personal meanings, and was based on an item each teacher brought to the workshop (Duffy, 2016, p. 381). By sharing personal values, a sense of trust and awareness for diversity was intended to be built. Furthermore, participants reflected and discussed *Images* by artist Jamie Wignall (Hallam and Gaunt, 2012, 85–87), exploring their teacher-student relationships throughout their biography. We then collaboratively explored the feedback tool *Critical Response* Process (CRP) by Lerman and Borstel (2003). CRP aims at supporting the development of any form of creative work through stimulating and activating feedback, led by the presenter. To actively engage networkers from the previous project, we empowered them to facilitate this process. CRP established learner-centered approaches to reflection, feedback and growth. All participants were engaged as responders, creating an open space for diverse perspectives, along with tensions and negotiations inherent. In CRP, teachers must engage in a critical examination of alternative and innovative ways of communicating. By not allowing participants to provide judgment or evaluation, they were confronted with a challenge to revisit prior opinions and sensitively choose words. Aligned with the work of Dweck (2006), as aforementioned, CRP focuses on what one is becoming, rather than on a deficiency-model. Related to CRP, we extended the opportunity of group feedback using *InterVision.* This method of collegial consultation evaluated by ICON (Innovative Conservatoire), is inspired by health-related professions (van Zelm, 2011).

As Lipowsky and Rzejak (2015) reviewed, using video sequences as a reflection tool in teacher training is considered an effective way to examine teaching practices and to aid in evolving, scrutinizing and developing teaching-related beliefs and attitudes. The reflection tool *Videography* involved collaboratively reflecting videotaped sections of instrumental and vocal music lessons using a four-stage process (see Table 1). Aligned with the studies examining the use of video-cases to promote reflection in preservice teacher education, we hoped that video-cases would encourage reflectivity and bridge a theory-practice gap (West, 2013).

**Table 1:** Reflection tool *Videography* with its four-step process.

1. Describing/What do you see?	2. Interpreting/explaining the meaning of the information or action	3. Personal opinions/suggestions for improvement	4. Personal implications/next steps for the presenter
The student did not talk during the lesson	The student seemed disinterested as he did not talk during the lesson.	He/She is a boring teacher as the student did not talk during the lesson.	In the future I would like to, e.g., ask more open questions to the students and vice versa so that the student will talk during the lesson.

Our considerations included ethical concerns or reservations teachers and learners could have about being recorded, and the selection, use, and storage of videos (Bucura and Kruse-Weber, 2021). We observed video recorded lessons by teachers outside the group. At the conclusion of each workshop, participants were invited to share their experience of the workshop in a *flashlight Forum*.

**Figure 5:**
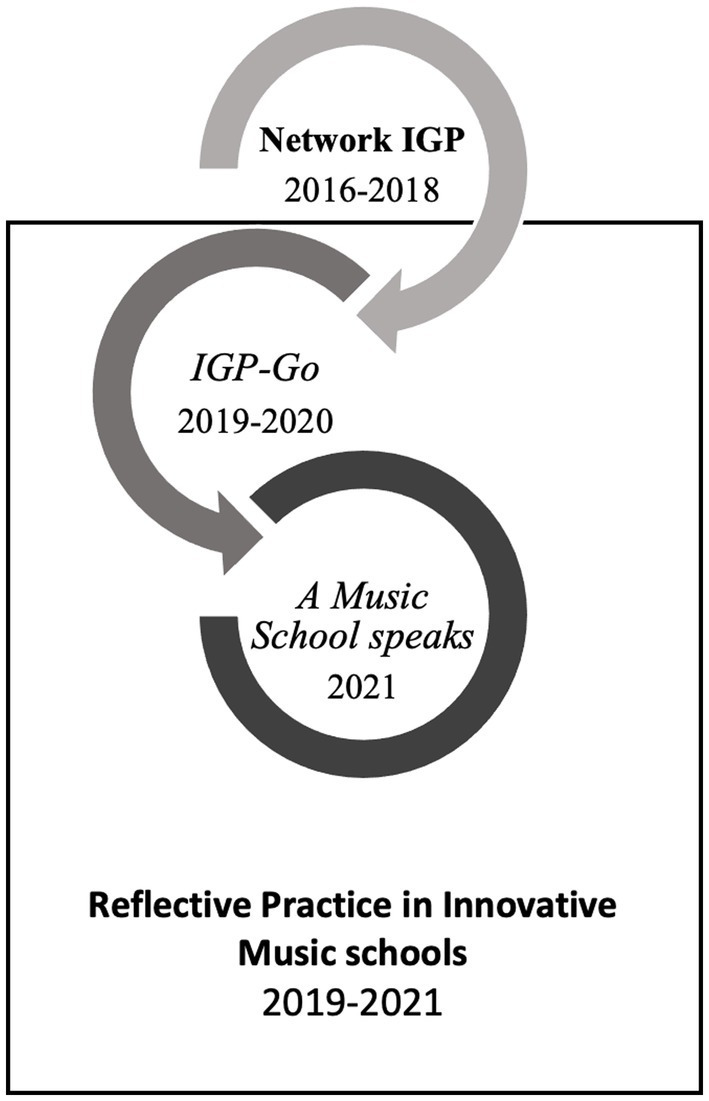
Cycles inspired by Design-based research.

Our research is influenced in its basic ideas by the design-based research approach (DBR; *cf.* Euler and Sloane, 2014). It implies the development of teaching designs, and a deeper understanding of associated learning processes as a theoretical goal. DBR allows the introduction of innovations and interventions in practice and to develop them in a circular process through research-based adaptation to the participants’ needs. Figure 5 points out the idea of circularity in DBR in our project: a first circle of workshops (Network IGP with 12 colleagues from the university 2016–2018) and second circle (Reflective Practice in Innovative Music Schools 2019–2021) as collaboration with one public music school (Kruse-Weber, 2018).^5^

### 2.2. Roles of the participants, researchers, and networkers

Given the complexities of this project, we clarify the roles involved. These include the research team, who—aligned with DBR—also intersected with facilitation and participant roles, teacher-participants, and the leader of the music school.

In order to recruit music school teachers for the project, we first explored interest in the project in dialog with several Styrian music school directors. We signaled that the teachers would be valued as people and not only as research objects, and that they would be actively involved in the research process, with space to tell their own story (Lambrechts et al., 2017). We explained that the project included implementation of innovations, which we would reflect, analyze and evaluate together. A director and trumpet teacher at a public music school in Styria (Austria), communicated openness and interest in the project. In January 2020, a project presentation took place at the music school; all teachers were invited. 18 teachers, including the director, attended the presentation as an engaged partner. We provided information about the study and protocols, and participants from the previous project described their personal experiences. Nine of the teachers, including the director, and five additional teachers that had not been able to attend, signed on. One participant held a double role: as teacher at the music school and at the university, where she had participated in the previous project. Over the course of the workshops, some participants attended regularly, whereas others came sporadically. For the seven meetings of Phase 2, a teacher that had not participated in the previous workshops joined the core group of seven teachers including the director and the double-role teacher. Demographically, teachers formed a diverse and heterogeneous group which contributed to the researchers’ aim of allowing diverse perspectives.

As shown in Table 2, the majority of participating music school teachers were female and above age 40. Most had more than 10 years of experience as music school teachers, five more than 20 years. Few of the teachers had a contract of more than 20 h per week. Teaching subjects varied widely: instruments, genre and teaching settings (one-to-one lessons, partner and group lessons). The majority of teachers taught more than one subject. Participants knew each other as colleagues, but to varying degrees.

**Table 2:** Music school teachers’ demographics.

		*Phase 1*	*Phase 2*	*Total*
Gender	Female	*n* = 8	*n* = 6	*n* = 9
	Male	*n* = 5	*n* = 1	*n* = 5
Age	30–39 years	*n* = 5	*n* = 0	*n* = 5
	40–49 years	*n* = 4	*n* = 2	*n* = 4
	≥50 years	*n* = 4	*n* = 5	*n* = 5
Music school teaching experience	1–9 years	*n* = 5	*n* = 1	*n* = 5
	10–19 years	*n* = 4	*n* = 2	*n* = 4
	≥20 years	*n* = 4	*n* = 4	*n* = 5
Employment at the music school	*Duration*			
	1–9 years	*n* = 7	*n* = 1	*n* = 7
	10–19 years	*n* = 3	*n* = 2	*n* = 3
	≥20 years	*n* = 3	*n* = 4	*n* = 4
	*Hours per week*			
	1–9 h	*n* = 5	*n* = 2	*n* = 6
	10–20 h	*n* = 5	*n* = 3	*n* = 5
	≥ 20 h	*n* = 3	*n* = 2	*n* = 3
	Teaching subjects^*^			
	Woodwinds	*n* = 4	*n* = 1	*n* = 4
	Stringed instruments	*n* = 4	*n* = 2	*n* = 4
	Voice	*n* = 2	*n* = 2	*n* = 2
	Brass	*n* = 1	*n* = 1	*n* = 1
	Drums	*n* = 1	*n* = 0	*n* = 1
	Keyboard instruments	*n* = 1	*n* = 1	*n* = 1
	Ensemble teaching	*n* = 4	*n* = 4	*n* = 4
	^*^ > one subject	*n* = 6	*n* = 6	*n* = 7

* Note that in this table, we included the participants who participated in the entire project, and excluded the ones that only participated in the first workshop (project presentation).

The two phases of the project (*IGP-Go* and *A Music School Speaks*) differed significantly regarding participant roles. In *IGP-Go*, researchers and networkers acted in a role Creech and Hallam (2017) described, as “midwives,” who were “enabling participants to discover the content and processes for themselves” (p. 64). As the research team, we supported or scaffolded participants’ learning, gave theoretical input about topics, and took into account their expressed interests. We also were careful about setting what we felt were challenging yet attainable goals when identifying/creating material and activities. In the second phase of the project *A Music School Speaks,* our roles resembled what Jones (2005) calls the “fellow traveler.” Fellow travelers empower the group for:

egalitarian relationships between leader and participants. As a result, the latter may feel more able to contribute their own ideas and sometimes will take on leadership roles within the group. The group may become a learning community, characterized by collective exploration (Creech and Hallam, 2017, p. 64).

We interacted with the group moving from cooperative (midwife) to autonomous mode (fellow traveler) in response to changing characteristics, dynamics and stages of the group experience. In the cooperative mode we guided the group by sharing ownership of decisions relating to the learning process. In the autonomous mode, we created conditions within group participants could take full ownership and responsibility for self-directed learning. Group members negotiated their own path with minimum intervention. The life experience and insights that all participants brought to the group appeared to be valued by the fellow travelers (Creech and Hallam, 2017).

In summary, the project team played several roles during the study. Influenced by DBR, we designed the workshops. As group participants, this also included contributing to group discussions, choosing and adapting material for the workshops, leading focus groups, making in-the-moment decisions about how and when to collaborate, and providing space for participants. Researchers met regularly throughout this process, discussing our roles and questions as they emerged.

### 2.3. Data collection and ethical considerations

Our data collection in this case study refers to the 18 workshops of *IGP-Go* and *A Music School speaks* (1/2020–10/2021). During all 18 workshops, group discussions were audio-recorded (three online and three hybrid), then transcribed, anonymizing speakers. In the first phase of the project, we recorded the focus group discussions from 11 workshops. In the second phase we recorded all seven work meetings of *A Music School Speaks*, as it was dominantly participant-led. In addition, participants were asked to complete a demographic questionnaire. All data were saved on a university server with safety precautions, including access restrictions to only the researchers. This study was closely coordinated with and supported by our legal department from the university, including an ethics board who advised the research team. Our considerations included the videography, which was complex, given the sensitive and personal nature of recordings involving teachers and learners. We invited and consulted an expert for this topic, and along with other stakeholders, organized a symposium with a discussion directly about it. This discussion was recorded and then analyzed by thematic analysis and led to in-depth discussions of storage, use, and consent with respect for others, with videographed lessons as data and/or learning materials (Bucura and Kruse-Weber, 2021).

Prior to the start of the group workshops, the participants signed an informed consent document and completed a brief demographic questionnaire on the online platform. They were informed that the workshops would be recorded and the data would be anonymized and used only for research reasons in this topic. The participants were also informed that participation was on a voluntary basis and that they had the right to withdraw at any time if they were not comfortable with the study.

### 2.4. Thematic analysis

The aim of the study and focus group discussion data led us to employ thematic analyses (TA; Braun and Clarke, 2006, 2021; Byrne, 2022). We regard TA as a “qualitative paradigm” (Braun and Clarke, 2021), in which we actively made decisions about the data. Accordingly, we were aware that our analysis is based in Critical Realism (CR), which originates in writings by Bhaskar (2008). CR distinguishes between the “real” and the “observable” world. This is understood that a material reality, which cannot be observed, exists independent from our ideas, and that our experiences and representations of reality are mediated by language and culture (Braun and Clarke, 2021). Nevertheless, it is notable that unobservable structures cause observable events and the social world can be understood only if researchers understand the structures that generate interventions. When we conduct an intervention, this establishes the conditions to create the intervention and we observe the results. CR allows us to look under the surface to an existence of independent reality to observe the underlying theoretical mechanisms and structures (Braun and Clarke, 2021, 286).

Accordingly, using CR we generated two types of codes. First, semantic codes, and second, latent codes. At the beginning we generated more semantic codes capturing surface meanings. Later, latent codes captured assumptions. We were three coders using “Consensus Coding” by coding agreements as a key measure of coding quality. We developed “a final set of codes through discussing which codes offer a best “fit” with, or provided a more accurate interpretation of the data” (Braun and Clarke, 2021, 285). All over, we took a reflexive approach to TA to account for researcher bias. Informal researcher memos helped us in coding sessions, primarily to define codes or summarize assumptions underpinning reading of the data. At times we revisited prior codes, further discussing and deepening our articulation of them. We employed the coding phases from Braun and Clarke, (2021) freely, meaning that on one side we approached codes systematically through the coding process. On the other side, we went back and forth to recursively generate the themes with clear concepts and to provide a distinctive telling of the story by checking a close connection back to other data. In all, we ended with 3,374 coding segments and 22 main codes (see Appendix Table 1). The code system was created in an iterative process that underwent several cycles.

Using the analysis software MAXQDA, manifold codes were generated line by line (Rädiker and Kuckartz, 2019; VERBI GmbH, 2022). Later, codes were sorted and grouped; where necessary, new broader codes or subcodes were created. Codes were merged and sometimes became enveloped as subcodes (Rädiker and Kuckartz, 2019). Collaboration took place mainly over virtual conference with all three researchers, yet occasionally in-person meetings were possible. Analysis meetings were held in weekly sessions over 1 years’ time, which allowed us to complete a thorough initial coding system. Afterward, we worked recursively as we reflexively considered new codes that emerged, leading us to return again and again to the first transcripts as we carefully reviewed each transcript in order to determine relevancy of new codes to all previous data.

**Figure 6:**
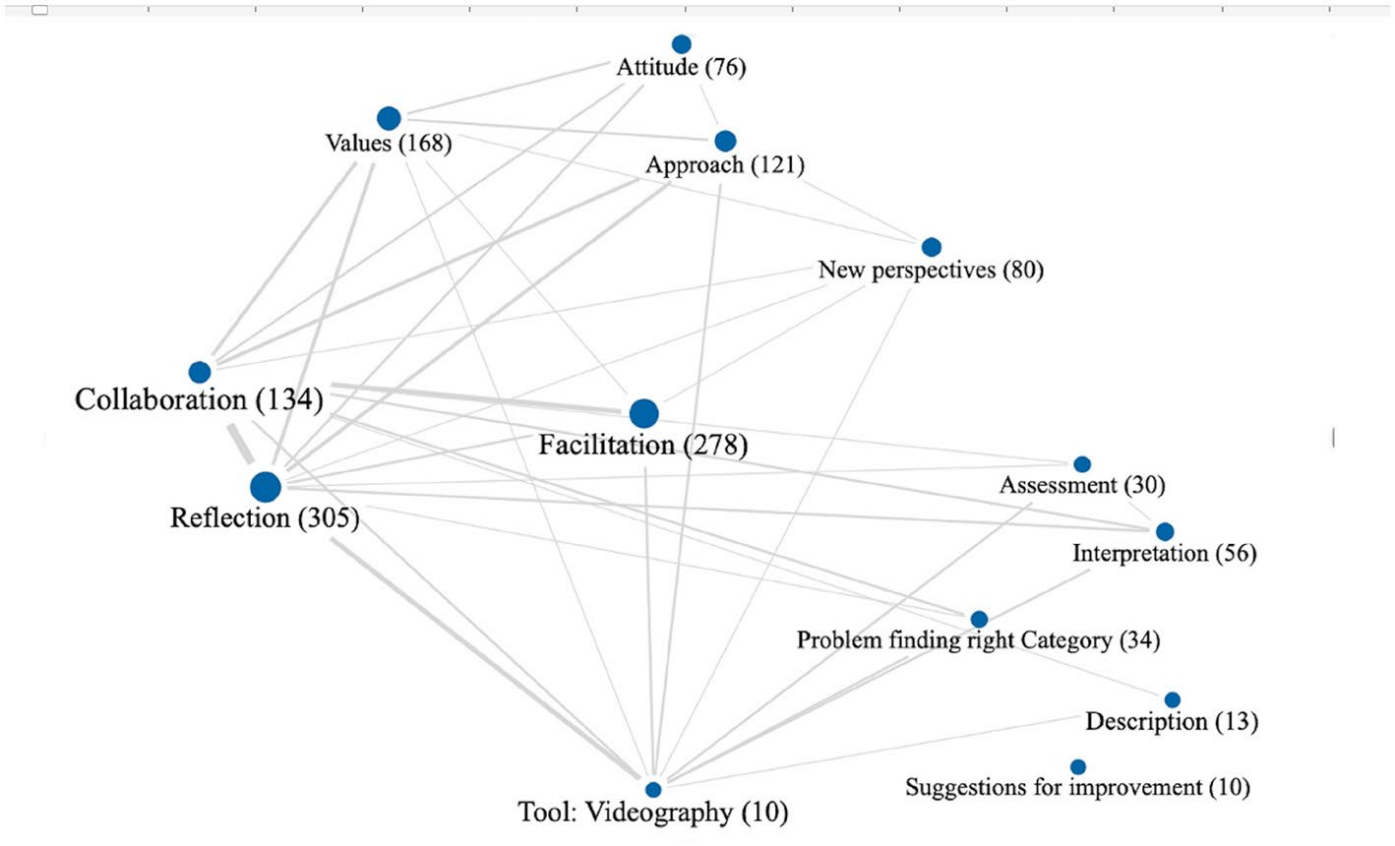
*Facilitating Videography*: Visual tool with relations of codes.

We then created summary grids and summary tables using the software MAXQDA (see Appendix Table 2), which allowed us to visually depict code recurrence and relationships, as well as to document emerging insights and explanations specific to particular codes, code groups and research questions. Additionally, we used visual tools from MAXQDA (see Figure 6). Each circle symbolizes a code, with the spacing between two codes reflecting how similarly the codes have been used in the data. This image shows that codes such as collaboration and reflection are very strong and belong closely together. The more overlap in one segment, the more they tend to have been used in the data material. Strongly connected are also the codes collaboration and facilitation.

After completion of all necessary coding cycles, we generated preliminary themes that had emerged both in discussions while coding, and afterward in a review of data. This process occurred to the point of data saturation when no new themes or insights emerged.

Further analysis (summary grids) referred to the code segments of our preliminary themes. Together, we sought to consider them openly, as long as the data could have adapted or changed them. In this process we revised the themes and this enabled us to specifically articulate them, sometimes combining them. We reflected the themes as a story with coherent data. Data are presented here in order to maintain participants’ voices whenever possible and contribute to study trustworthiness. Although workshops were held in German and are documented here in English, translation was carried out with care. This process was long and in-depth, often leading to rich discussions among researchers about not only definitions, but ways cultural understandings may imply specific use of a particular term or phrase.

## 3. Results

Upon an in-depth, collaborative coding and analysis process, we considered the literature in relationship to the research questions. As a result, the data led to five themes and several sub-themes.

- Group cohesion, inspiration, and appreciation of collaboration
- Bridging and transferring theory and practice
- Deeper thinking with teachers as learners
- Challenges and potentials during the COVID-19 pandemic
- Finding the music school’s identity and value

### 3.1. Group cohesion, inspiration, and appreciation for collaboration

#### 3.1.1. Appreciation of collaboration

In this study, participants expressed appreciation for the opportunities the workshop series presented them. They regarded them as important social and professional time. Participants noted the value others’ perspectives brought to them, and often remarked about their own need to connect with others professionally. While the workshops indeed had a professional and purposeful tone, time was also granted during which participants informally connected, for instance at gathering times and breaks.

Participants remarked the group felt cohesive. This sense of togetherness likely provided them strength to overcome hindrances, for instance tiredness in the evenings. Furthermore, small groupwork with 3–4 people seemed to be a powerful context for learning and negotiating. They noted they felt secure. After small group work, participants shared their experiences with all.

I came here, like many others, straight from class, tired, a bit groggy, I thought, well, but I have to say it was great, and especially the work in the small group, I enjoyed it very much (…).” (IGP-Go:1)

Participants expressed missing professional exchanges in their daily lives and noted they regretted such exchange did not occur naturally as they felt it had years prior. They stated:

So, I find it rather tragic that we have such a workshop in order to have an exchange. (…) I’m horrified to realize that I’m about to enter my fortieth year of teaching and there have been changes … this sticking together happened automatically in the past (…). (IGP-Go:1)

Participants’ interests were also social. Many commented on personal experiences and all appeared to be listening to one another with attention and respect. These factors seemed to create an atmosphere of trust, furthering social ties. The group did not know one another well, and stakeholders remarked it was “fascinating” when they got to know colleagues and their perspectives. One noted they were surprised by colleagues’ choices of representative objects and by the stories behind them.

And when I have such a short talk with colleagues today, where you think you know them, (…) and then you don’t know them at all, (…) it surprises you what he has to tell and that’s what I find exciting (…). (IGP-Go:1)

Participants commented on the trust they had built throughout this project. They noted that they could be vulnerable, taking risks, being “wrong,” and failing together. Moreover, they missed their colleagues during the COVID-19 lockdown, and remarked that virtual meetings were valuable to them in allowing consistent professional connections during an otherwise isolating time. Some even expressed pride in being involved in this project. One said:

I’m also very happy about the project … I’m almost a little proud that we have this at the school. (IGP-Go:2_2)

The appreciative atmosphere in the meetings seemed particularly poignant when, during the project, the COVID-19 lockdown necessitated meetings be held virtually. One participant indicated that seeing one another, even virtually, was welcome amid distractions of managing their personal and professional lives during this uncertain time. They said:

I’m trying to manage somehow (laughs) between, if I can ever get access to a computer that works, because of course everybody in our house needs one, between learning with kids, taking care of students and other stuff. Yeah, (…) glad to see you guys (laughs). (IGP-Go:3)

A driving factor that bonded the group appeared to involve quality of communication. Participants said they appreciated communication with the university. They enjoyed the dialog, specifically on “eye-level” with university colleagues. The non-hierarchical and direct line of communication between institutions seemed particularly important to participants in that they might align their values and create complementary systems. Another indicated their appreciation for the ways workshops were purposeful and facilitated, noting it created a valuable space for dialog. They stated:

I have had such a nice group, (…) and stimulating exchange, (…) I have also led a music school for almost thirty years, and such an exchange of ideas, such an intensive engagement is simply not possible in everyday business (…) To find the time and peace where topics are given and moderated, that has a different quality. (IGP-Go:1)

The use of video reflection seemed challenging for the group. See Figure 6 with the visual tool, there was a strong tendency to interpret or even evaluate, rather than describe what they viewed. The task, however, seemed to nudge participants to think differently about the lesson they viewed. When they leaned toward evaluation or judgment, others noticed it and the group steered themselves with good humor. These challenging tasks appeared to strengthen the group. One participant described it:

So, (…) for me it’s very good that I am able to be descriptive, which I find very, very difficult. We are not trained that way. We are immediately trained to interpret. (…) Describing is, I think, a very important tool, because it helps us to look at the content. (…). (IGP-Go:2_2)

Several teachers expressed that in their daily professional work there was typically little opportunity for an in-depth exchange of professional issues. One participant stated this was exacerbated in recent years because lecturers had only part-time contracts, indicating they may not encounter colleagues as often or have the same time to build relationships.

In our analysis, a prominent code was appreciation of collaboration. As presented in Appendix Table 1, collaboration was connected to topics such as values, attitudes, approaches and new perspectives. Participants used words such as “exciting,” “improvement,” and “pleasant and enriching” when describing collaboration. One remarked they were affirmed when other colleagues commented in alignment with their own thinking, and others noted their views had been broadened, that they felt self-reflective, and could improve and develop their own thinking. Another emphasized the importance of trying something new. They noted, however, the difficulty of making time.

While many participants commented on the importance of growing their perspective and hearing different views, some were more specific. One participant noted that the collegial exchange provided them with a chance to view students in different ways, as well as to regard their own communication with students.

I also really enjoy the (…) professional exchange among each other (…) that is simply an immense enrichment, (…) this is benefiting me a lot in regard to the teaching practice lessons, where you have to deal with students, (…) and where you, (…) get a different perspective on students, that has become clear to me today (…) I appreciate it very much. (IGP-Go:7_2)

In using Critical Response Process (CRP), the stages were not meant to be followed through or be used as distinct categories, but to stimulate discussions and raise awareness of different views. As noted, it seemed challenging for participants to think within categories in the four-step process (34 codes) of videography reflection (see Figure 6). For instance, describing situations seemed to be difficult (13 codes), while interpreting (56 codes) and evaluating (30 codes) appeared easier, occurring more often. The purpose of these steps is that descriptive feedback fosters learners’ own interpretations. Participants commented on numerous aha-experiences to this point. After some practice we noted that the participants became increasingly careful and began to correct themselves when they felt they had interpreted or evaluated too quickly. Participants seldom gave suggestions for improvement about others’ teaching videos, but they expressed appreciation for this process. One said:

Yes, (…), interpretation of each video by (…) the colleagues is just really enriching every time that we exchange and for me especially self-reflection comes up again, where I position myself in this extreme “passive-active teacher,” and how one switches back and forth at best. (IGP-Go:7_2)

#### 3.1.2. Group decisions

The upcoming conference date with the presentation *A Music School Speaks* necessitated the group efficiently make decisions, not only on presentation content, but also practical matters of organizing themselves. Some took on leadership by organizing the group. Everyone from the task force expressed a need to contribute their own work.

#### 3.1.3. Expanding traditional conceptions, emphasis on inclusiveness, and accessibility

Participants regarded their students not only as musical learners but also as clients seeking music therapy and wellbeing. Over time, the group began including more adults and seniors in their visionary concept. They considered methods, tools and approaches of music therapy to create learning environments and to facilitate learning for individuals with disabilities.

### 3.2. Transfer in bridging theory and praxis

Participants declared not only interest in applying new ideas to their teaching praxis, but had ready and willing attitudes, for instance, “I definitely will do that!” Transfer in applying a theoretical idea however, appeared to be considerably more difficult. There seemed to be a willingness to entertain new ways of thinking, yet less assertion in considering how and in what ways to apply theory to their practice. They had complex perspectives. For instance, participants remarked about bettering their understandings of the science behind music pedagogy, their interest in better understanding not only how to teach music, but why they taught the ways in which they did, and how to better reflect on their own common-sense knowledge toward a more purposeful practice.

#### 3.2.1. Appreciating connections between theory and practice

In this study, participants expressed wanting more connection between the content at the university and their own job demands at the music school.

And then I realized again (…) we should do more theory or less theory; I think to myself: Why do you always have to separate that so strictly (…). (IGP-Go:11)

They remarked that they tended to associate the university with theory, while to them the music school represented praxis. This dichotomous way of positioning theory and praxis appeared to be widespread among participants and they said they had neither considered the different relationships between theory and praxis, nor had questioned which might follow the other. One participant remarked:

For me, the first question that came up was, does practice always have to follow theory, and the way you’re planning on doing it now, to turn the whole thing around a bit, to actually move from practice to theory for the next generations. I find that very exciting and important for our work (…). (IGP-Go:1)

Participants expressed their interest that not only content from the university came to the music school, but that knowledge and experiences of the music school should transfer back to the university. The teachers expressed their desire to see this type of mutual exchange and told us of their appreciation for the project toward these possibilities. Participants said they valued openness in the group, having space and time for exploring new ideas, and university colleagues’ willingness to learn from their experiences. One participant said:

(…) when I came back from university, I was full of knowledge, and then the first thing I saw in class was a seven-year-old child who was learning the recorder with me, and then I said, “one more day like this and I’ll quit.” Because I just didn’t know what to do with the child. (…) your interest is really to go to the base and see what’s really happening there, and then to bring that to the university (…) students come to the music school and engage with that, because then they are close to practice, so to speak. And I think it’s great that you’re doing that. (IGP-Go:1)

#### 3.2.2. Prioritization of theory and practice

One participant felt that more praxis should have been part of their university studies. Others remembered teaching practice with their own students in the higher education setting as exhausting, yet acknowledged it made the transition to professional practice much easier.

[In music education training at the conservatory] I had (…) to prepare a lesson every week. At the university you do a sequence once a semester. (…). As a student, that was already an insane challenge for me, when you suddenly have to prepare a complete lesson, week after week. (…) Of course I was happy, after the year I was able to teach (…). (IGP-Go:2_2)

Participants interestingly noted their widespread perceptions of a gap between university preparation and the job of being a music teacher. One participant noted that the realities of their job looked different than could be replicated at the university level with preservice teachers. One said:

(…) Well, I think, there is quite a big discrepancy between what the students hear in the pedagogical courses, for example, what we have heard (…) at the university, (…) practical life often looks a bit different, but this is a long way to go (…). (IGP-Go:4)

### 3.3. Deeper thinking with t(eachers) as learners

Participants appreciated gaining new ideas in the group, giving the chance to broaden their horizon and to deepen their knowledge. This point appeared to relate to all aspects of the data. One participant said:

To get new input, new ideas, how colleagues that have been teaching for decades, still have a very fresh approach to the whole profession. I find that very impressive. At the same time, getting new ideas that broaden one’s own horizon, that I found very, very good. (IGP-Go:1)

#### 3.3.1. Reflection tools as inspiration

The workshops seemed to provide space to try ideas out, get them wrong, and start anew. Participants expressed a need to articulate and to consider what values might be important for the music school now and in the future. Thus, in this study teachers also became learners.

But I think why the exercise [a reflection tool] does so well is to force us to think: What are our values, actually? (…) Because there are values that we might share more or less as a society and there are values that are more important or less important to us personally. And I think it’s totally important that we are aware of our values, so that we can differentiate once again. (IGP-Go:8)

Participants also noted the benefit of getting to know new teaching and learning approaches, values, and attitudes of their colleagues. Participants said they sought opportunities to learn from different instrument groups as well. One said:

It was (…) exciting to learn something about the other instruments and then to see, okay, we have basic topics, we have a lot of the same topics, where we then (see) that there are different circumstances with other instruments, but it’s just exciting to get into the details in this way. (IGP-Go:1)

#### 3.3.2. Participants’ language

During data collection, participants’ language appeared to converge. Some seemed to develop what we referred to as problem awareness for language and as a result, seemed to become more sensitive to concepts of teaching and learning. This included, for example dealing with students, teacher-and learner-centeredness, and mediating learning content. After repeated sessions participants began using similar language and more readily agree with one another’s use of it, or in some cases assume others’ meanings as agreed upon. Meanings seemed to result from their earlier discussions and use of the reflection tools. Common language also seemed to lead to a more specific articulation of themselves as music teachers, and of their profession. This point was also identifiable in the use of pictures and video, as participants noted that everyone sees and hears something unique. One reflected:

I have to agree with (a colleague). I felt the same way, same images, same situations, but the different ways of thinking, the different ideas that everyone has about it, that was exciting. (IGP-Go:2_2)

### 3.4. Challenges and potentials during the COVID-19 pandemic

One big challenge for the project and participants was the COVID-19 pandemic. They expressed their experiences, both personal and professional, which included the objectives they had for online teaching, possibilities in communication, technical issues, time management, and ways they balanced the positive and negative sides of pandemic-related changes.

#### 3.4.1. Personal situation of everyone involved (participants, students, parents)

All participants remarked that time spent on computers had rapidly grown during the pandemic. As a concept, time had eroded. Despite these challenges, participants also noted positive outcomes. People and families were brought together, for example providing time and space for making music.

Several participants experienced challenges in fulfilling different roles, such as being a teacher, parent, homeschooling their own children, and managing household tasks. Participants said they wished to separate work and private life, but could not achieve it. Several participants said they suffered from isolation during the pandemic, including in their work because they no longer were “feeling good” after a lesson with colleagues to share or discuss with, take breaks with, and in general replenish one another. Teaching without these boosts left many feeling drained.

I think the common breaks have always been important (…) and that’s all missing now. Even if some are at school sometimes, one stays just in one’s room (…). One doesn’t see and hear anything, and there [in the breaks] we could forge joint plans again, somehow, and new ideas could emerge. (IGP-Go:11)

The director was also challenged during this time, and said he often felt overloaded with the responsibilities to communicate official rules to the teachers and parents. He said he needed to both balance and continue to work as normally as possible while supporting the teachers, for instance by encouraging them to care of themselves.

During the time of initial lockdown in this study, teachers realized they benefitted from increased insight into students’ home lives. This was helpful in understanding how the students’ homes were equipped for instrumental and vocal practice (or not), possible disruptions that might be factored into practice strategies, and so on.

#### 3.4.2. Teaching (online) objectives

Objectives for teaching online were one aspect of their careers that participants agreed needed to be rebalanced. In this study, the general attitude of the participants was to stay in touch with students and parents during the pandemic. They valued this communication highly and as more important than improving the student’s instrumental/singing skills. They encouraged their students to record videos of themselves playing or practicing to send to relatives, which they viewed as motivating.

#### 3.4.3. Technical issues and communication

During this time an additional challenge included technical issues, such as unreliable internet connections, knowledge of equipment (hardware and software), and best practices for audio recording. Technical issues seemed like the most challenging efforts for participants. With blurred lines of home/school/music school, participants sent additional materials to students to implement in practice sessions, for instance WhatsApp and email messages, and students sent in practice videos. One participant said online teaching can be helpful in promoting increased home practice, for instance working hard to get one’s recording just right. At the same time, participants recognized that online teaching lacked an atmosphere of togetherness and direct interactions (also musically). Traditional individual lessons were overwhelmingly favored by participants.

I agree with (a colleague’s statement), I miss the personal contact and so do the children. Phone calls are very exhausting in the long run because of the often poor connections. (IGP-Go:4)

### 3.5. Finding the music school’s identity and value

When participants needed to collaboratively settle on details of their own presentation for their music school vision, they seemed to take on deepening ownership. The role of the director seemed integral to this, while members took on new perspectives. Through this process they began to articulate and contextualize their views of teaching and learning, and they were able to identify a shared philosophical rationale about the power and importance of music. One participant described new roles.

For me, when I’m listening to you (Silke), it is a bit like supervising, because up until now we have really worked, collected thoughts, and brought in different aspects. Now we get supervision. I think that’s great. This view helps us to better recognize what we are actually doing. (EMS:1)

#### 3.5.1. Role of the director

The director walked the line between providing leadership presence, while also stepping back to maintain space for others. He claimed they had grown through the process and noted a shift in perspective. He said, “The vision *A Music School Speaks* has opened doors for us toward viewing our school in a new way, with approaches that we more or less knew before” (EMS:1). Participants hoped that their presentation would inspire other colleagues.

#### 3.5.2. Taking ownership of new perspectives

In the second phase of the project, the task force of teachers attempted to contextualize their job and their school in relation to the broader field of education. It took much time and investment for the group to get to the point of answering their own questions. Original aims of the project involved realizing and acknowledging one another in order to agree upon a common goal. In this process, participants became more sensitive about their language, content and communication. The title and main mission for justifying their identities such as music teachers and as institution became: “that’s why we are important.”

Participants discussed the purpose of their music school at length, indicating their regard and value for the role it played in the social and cultural life of the greater community. While describing their visions of music school some noted the importance of the music school space as a meeting place in which to be social and neutral. They mentioned the café and performance spaces as well as art displays. One aspect of the school they felt made it unique was the flexibility of their spaces, which included moveable walls and multipurpose rooms. Ways in which the space lent itself to a variety of needs–and might even inspire them–felt particularly special to the participants.

Our building is located near the school center as well as the cultural center. It reflects the pedagogical concept in terms of integration, inclusion, diversity, flexibility, climate neutrality, transparency and self-determination. (…) It is not quite complete, perhaps. It is a place to stay and meet, a communicative, transparent social space. (EMS:5)

When looking to the future, participants had many ideas. In general, they indicated an interest in becoming a pillar of their community’s social and cultural life, thus necessitating openness and outreach to all people in the area, including and beyond those the institution already served. They hoped to expand the range of ages represented at the school with more adults and seniors, as well as young children, in order to be inclusive. One remarked:

One idea we had was, to no longer call the school a school, but a music competence center. (…) A large reservoir with music theory, music therapy, with adult education, a library for sheet music, so that people can come here and say “I would like to have this sheet music or this song, do you have it?” (EMS:1)

As the study went on, senior citizens were discussed at length, as the group realized they could benefit much more from music-making and may have the time and inclination to become involved. One participant reflected:

I am a folk music teacher (…). I take my guitar and then we do folk singing. Actually, they flourish. The seniors are so satisfied. You go home so satisfied when you see the radiance in their eyes. (…) That should be done much more, then people are healthier and not so alone. (EMS:1)

Participants noted that new populations of people might feel hesitant to participate. They wished to provide low-risk opportunities for people to come. They also sought to foster cooperative relationships with local clubs and to provide a range of possible days and times to accommodate different schedules. One described the vision like this:

There is no fear of entering our institution and people are happy to make use of the teachers’ know-how. So we open our doors widely and bring people into our house. On the other hand, we also reach out and work in multiple cooperations on a network of music (teaching). Our music school is integrated into the regional education network. Teachers from the different schools work together in a spirit of mutual appreciation (EMS:5).

#### 3.5.3. Contextualizing

Over the workshops, participants began contextualizing not only their work among and in relation to one another, but also in a broader professional sphere. This included situating themselves in their city and region, in their country, and in their language and cultural values. They began acknowledging that characteristics of the music school and students were not the same everywhere, even in their own region. We noted a growing awareness of themselves in relation to a broader, diverse field of practice. Such comments indicating a growing sense of their ability to situate themselves culturally and socially also included considering themselves among other music teachers, for instance in other music schools and those who worked in public schools, as well as their relationship to local and regional university music pedagogy programs and curricula.

When looking to the future, participants agreed on the power of music, often providing personal anecdotes to support it. They agreed on the value of music and had different rationales for why this might be so. One participant noted a refinement of humanity they felt could be enhanced through musical participation, stating:

But I think our mission here is (…) we simply offer to young people, older people who visit our music school, that they are refined on their life path, in their thinking. That they can work creatively, can develop and live out creativity, that they are educated to be thinking people. I think that is our mission here, that they can refine their being human here (EMS:1).

## 4. Discussion

We examined a CPD project with instrumental and vocal music teachers. The purpose was to deepen our understanding of the situation of instrumental music teachers, including their work, approaches, attitudes, experiences and receptiveness to growth and change, as well as problems or barriers to knowledge transfer. In the following section, we discuss and interpret these questions in relation to the data, themes and literature.

### 4.1. Expanding professional development and participants’ experience

Professionalism involves ethical questions that concern who we are and who we want to be as music education professionals in today’s society (Westerlund, 2017). Together, participants reconsidered these questions in the first part of the project *IGP-Go*, and they provided a central tenant in the second part of the project *A Music School Speaks.*

#### 4.1.1. Forming group cohesion

A major tenant in the professional development process had to do with the group coming together as a safe, trusted community. One of the ways to achieve a grassroots transformation was through careful and non-hierarchical facilitation, based on mutual communication among all. It was important that participants could tell their own stories (Miksza and Berg, 2013), and express their own thoughts in an atmosphere of trust, appreciation and openness (Williamon et al., 2021). This facilitation provided role-modeling for respectful and appreciative interactions, as well as reflection about oneself in relation to others’ experiences. Participants accepted responsibility for themselves and formulated a personal, and increasingly unified professional vision for themselves (Hammerness, 2006a). This was in stark contrast to what many felt were imposing governance made by politicians (Westerlund et al., 2019).

Data suggested that reflective practice and collaboration were valuable to participants. CPD required all stakeholders engage in many ways including what Fraser (2019) describes as developing new skills and understandings, taking on extra work, risking failure and inviting possible disapproval from staff and students. Data indicated participants experienced a supportive and creative environment that enabled positive interactions and mutuality (Creech and Hallam, 2017). As Gaunt and Westerlund (2013) noted, such an environment can promote innovation and negotiation of cultural differences and meanings. This is aligned with Georgsdottir et al. (2003), who emphasized ripe situations: if “the environment is not ready for creative, innovative ideas then original thinking will not flourish” (p.184).

Participants’ discussions, including verbal descriptions, reflections about experiences, attitudes, and expressed beliefs, provided insights to one another about music lessons at their music school. This made sense to us because as Jørgensen (2009) noted, teachers must discuss, explore, and reflect qualities of teaching and learning with others. We felt strongly that effective support for teacher learning was furthered by an emphasis on dialog and collaborative interactions (Reynolds et al., 2010).

The power of group cohesion in the second portion of the project made it possible that all seven participants were able to perform and present their jointly-developed vision of music school work. They did this as a team and referred to themselves as a task force. The participants’ self-declared title *A Music School Speaks—That’s Why We Are Important* intended to offer a fresh perspective on justifying music education in publicly-funded music schools. It implied to us the group’s confidence and highlighted empowerment characteristics outlined by Kirkman and Rosen (1999). It also seemed to reflect some degree of self-determination, in particular the autonomy and relatedness participants gained through participation (Ryan and Deci, 2020). This was apparent in an identifiable flexibility and resourcefulness (Bucura, 2020a). They also appeared to gain a sense of competence as they dove into theoretical and practical discussions, fine-tuning group values and a sense of togetherness (Ryan and Deci, 2020).

#### 4.1.2. Addressing pandemic challenges

Another aspect of the professional development process included the COVID-19 pandemic. The teacher-group had different perspectives in dealing with music lessons remotely during lockdowns. The findings complement studies, for example, of Biasutti et al. (2022), Camlin and Lisboa (2021), and Schiavio et al. (2021), regarding a range of challenges, responses, difficulties and positive experiences for instrumental teachers during this time, such as lesson planning, time management, student involvement, and communication technology.

Psychological implications of the digital “turn” were apparent in multiple roles of teacher and parent. Finally, the reflections highlight the pressures this crisis placed upon educators to adapt swiftly to technologies while maintaining high pedagogical standards (Schiavio et al., 2021). Many mentioned aspects of responding to this pandemic (and change), which can be summarized as a core competence of flexibility (Georgsdottir et al., 2003). This has a strong impact on teacher training courses as we must address and encourage flexibility for pre-service teachers.

### 4.2. Dealing with the reflection tools

The second research question involves ways participants collaboratively discussed using reflection tools and ways they may have identified with workshop interventions. Reflection tools seemed to play an important role in participants’ abilities to embrace different perspectives. The tools provided a lateral space by which participants could lend an ear to new possibilities not previously considered. They allowed participants to be open, mediating their growth differently than hearing from someone else with a different viewpoint.

#### 4.2.1. Bridging theory and praxis

Our intention with the reflection tools was to critically reflect their potential for teaching and learning by mediating “between experience, knowledge and action” (Gray, 2007). Critical reflection promotes consciousness and hence the potential for autonomy, allowing informed judgments, and critically examining underlying assumptions.

Accordingly, participants critically discussed both potential benefits of the interventions for reflective practice (e.g., Critical Response Process, or the video-observation phases, and potential challenges of a student-centered approach). We sought to shed light on responses to the reflection tools. With the Critical Response Process (CRP), we intended to connect ethics to practice, considering possibilities of ambiguity, empathy and diversity toward building and furthering a participation. As mentioned, in the first step of the critical response process learners expressed overall statements of meanings (Lerman and Borstel, 2003).

Feedback was presented as a “foundational aspect of meaningful reflection” (Lerman and Borstel, 2003, p. 4) The CRP allows to consider teaching from different perspectives (Legette and Royo, 2021). The biggest difficulty for all participants in the group seemed asking open questions of learners or presenters. Regarding feedback from students, they were not accustomed to it and often felt unsure how to respond. According to Sandars and Murray (2009), reflection (i.e., asking questions) can be learned. Participants suggested developing tools for pupils so that they have more possibilities to express their experiences in music lessons and ask questions.

As aforementioned, the terms theory and praxis are often used dichotomously (Niessen and Richter, 2011), but this hinders professionalization, then becoming integrated only slowly if at all (Kruse-Weber, 2018). The profession of instrumental music teaching tends to lean toward traditions like a master-apprentice model (Bennett and Hannan, 2008), unlike other types of educational institutions (Gaunt, 2016). There is a gap therefore, between educational theory and practice (Kruse-Weber, 2018). In this study, participants indeed tended to dichotomize theory and praxis, also applying institutional labels to them, for instance, university represents theory while music school is “real life” praxis. It appeared to be eye-opening to the participants in this study, however, to consider a deep, reciprocal relationship of theory and praxis and the ways it might help them not only reflect on teaching, but also express their values. These considerations can also affect institutional relationships, as dichotomizing the terms may not breed the kind of cooperations that Darling-Hammond et al. (2005) noted are lacking. Yet, participants in this study valued communication with the university and the opportunity to align values, curricula, and vision, which authors emphasize is important (e.g., Hammerness, 2006b; Grossman et al., 2009; Bucura, 2013; Bucura, 2020b; Bucura, 2022).

#### 4.2.2. Finding a music school identity and value

We recognized the process to strengthen identity is dynamic, interactive, and ongoing. Wenger (1999) noted that communities of practice may involve people sharing concerns or passions, and that they can improve these communities through regular interactions. This seemed to ring true in our study. Participants had ample time together, not only in each workshop, but in many workshops over a lengthy period of time. The COVID-19 pandemic likely also played a role in further bonding the group as noted in the prior section. Despite moving meetings online, individuals faced new challenges like isolation, yet continued to connect regularly with one another.

Participants built a shared repertoire of not only resources and practices, as noted by Miksza and Berg (2013), but also language, agreed upon values, and group norms that seemed to not only solidify them as a group, but to provide a sense of personal and professional identity. Data indicated the group’s growing sense of themselves, particularly through their language. In the beginning, it seemed participants may have been speaking different languages in that they often needed to clarify their meanings. Over time however, the group appeared to become more comfortable, better articulating themselves, and finding easier points of understanding.

### 4.3. Developing participants’ thinking, attitudes and definition as a group

The third research question addresses participants’ potentially changing thinking and attitudes, and ways they defined themselves as a group. Education should empower learners with skills and competences, particularly to address continual changes (Herodotou et al., 2019). As learning is socially situated (Lave and Wenger, 1991), workshops in this study seemed to provide Renshaw’s (2009) descriptions of facilitation—as a non-directive way of engaging a conversation in order to empower others’ ownership.

#### 4.3.1. Facilitation of deeper thinking

The theme “deeper thinking” is in accordance with the studies from Lipowsky and Rzejak (2015), as the depth and quality of teachers’ content-related reflections also seems to be important for their development of competencies. Deep learning outcomes, as noted, can be defined as those that collectively comprise understanding, and the ability to apply that understanding, rather than memorizing or imitating (Marton and Saljo, 1976). A change in teachers’ beliefs can be predicted by the depth of content-related processing. Nevertheless, lack of time – often stated among participants – is considered as a barrier for reflection, innovation and interaction (Carbone et al., 2019, 1,358).

As Feiman-Nemser (2001) noted, professional discourse such as discussions, can help to deepen subject knowledge, refine instructional repertoire, hone inquiry skills, and build critical thinking among colleagues. Participants in this study indeed appeared able to deepen their perspectives in these ways throughout the course of their participation. This seemed especially apparent in critical thinking, as participants questioned one another and themselves. For example, they increasingly articulated needs for adult and senior populations, as well as students with disabilities. In this way, participants took responsibility while re-thinking, re-questioning and re-positioning music school issues and challenges from politics and society. One of the tools used, a vision of a lighthouse, seemingly inspired them to “open doors” and consider the music school in a new way.

Gaunt and Westerlund (2013) refer to cooperative and collaborative forms of learning as one of the most powerful ways to constructively deal with current challenges and the dynamic developments of professional practice. As also shown in Figure 1 this is aligned with our findings and our experiences in the workshops (Kruse-Weber, 2018). Collaborative exchange among colleagues would have a great potential to drive learning and teaching in music (high) schools, which also benefits professional satisfaction. Finally, the findings of music pedagogical theory find their way into practice more easily, and vice versa, when experiential knowledge of practitioners is incorporated into music pedagogical theory (Kruse-Weber, 2018).

#### 4.3.2. Facilitating empowerment

Verhulst and Boks (2014) emphasize empowerment as a major aspect of giving authority to a learning group; this includes power, decision-making, responsibility, and strengthening self-determination, creativity and autonomy as well as fostering (educational) knowledge and skills. Growth and motivation are supported when the leader shows engagement and provides clear accountability towards others who will be affected by change (Adams, 2003; Carbone et al., 2019).

Many participants initially contributed to group discussions with a focus squarely on practical elements of their teaching approach, and often a firm declaration of their teaching philosophy. Researchers note this is not uncommon, particularly among early career music teachers, whereas experienced music teachers may increasingly consider other factors important, like their overall impact on those they teach and their contribution to the profession (Baker, 2005). As workshops went on however, participants encountered different tools and discussions involving not only different views, but considerations for outsiders’ perspectives, both colleagues and others.

#### 4.3.3. Developing resilience and expanding inclusion

As Westerlund et al. (2019) describes, the music school developed some pathways to transformation and inclusivity in their school through the power of music. For instance, the group was expanding traditional conceptions of music therapy and music education. Westerlund spoke of these complimentary fields:

(…)on the one hand, by setting goal-oriented music learning at the heart of music therapy, and, on the other hand, by using the expert knowledge of music therapists to make music pedagogy more inclusive and accessible (Westerlund et al., 2019, p. 21).

It is important to note that the group emphasized that *everybody* should have access to the music school—free-of-charge possibilities should be possible. In line with this, the music school presented itself with “open doors” for all people throughout the city.

## 5. Implications and conclusion

Conway (2008) points out the desideratum of research in professional development activities and success from (instrumental music) teachers. Therefore, this study provides only one contribution regarding professional development. We note that we did not yet have the possibility to reflect and discuss the experiences of the participants after the project. The subjective impact for each participant is yet unclear. Nevertheless, as teacher training programs mostly consist of complex components it is often not feasible to identify single features responsible for the effectiveness of a positively evaluated training program (Lipowsky and Rzejak, 2015). In further analysis we should follow the development from each stakeholder in the project. Future studies should aim to understand and sensitize for the four steps involving teaching video feedback, as our participants did not share their own videos.

Some qualifications should be mentioned. Had researchers not participated in the workshops the data could perhaps have differed, however participation roles contributed to rapport. While the project spanned a lengthy amount of time—nearly 2 years—we considered this a study strength because all data were painstakingly documented and reviewed, and time existed to reflect on data analysis.

Implications of this study indicate necessary conditions for meaningful collaboration toward instrumental teacher professional development. These include significant time and continuity over time in order for members to negotiate a group identity, along with a sense of trust and safety. Like the participants in this study, it appears important to build a sense of shared identity with collaborators as well as to contextualize one’s work within a broader profession of music teaching, including other music teachers, other places, languages, cultures, and regions, as well as toward university programs with which it works. Facilitators should provide tools for thinking and re-thinking pedagogical approaches, while providing ample space for expressions of participants’ own meanings, realizations, misunderstandings, questions, and discussions (including disagreements). Trust is essential for such meaning-making, and facilitators can do much to enable it by making space to talk, eye contact, nodding, and other expressions and gestures of interest and engagement. Tools can provide principles for considering new perspectives in safe ways. In this study, diverse perspectives were welcome and necessary. *Videography* served to build holistic thinking while sensitizing perception and consequences of describing, interpreting, and judging video-recorded lessons. Increasing prominence of digital content necessitates these considerations for teachers. Virtual workshops may necessitate different considerations to foster a community of practice than hybrid or in-person. Ethical considerations for video use are relevant to additional fields that use video-recordings as a didactic resource. Notably, this applies in general teachers’ education, as pedagogical considerations are central, but concerns of sound quality and artistic interactions are likely lessened (Bucura and Kruse-Weber, 2021).

Similarly, it is important to recognize that video recordings offer the possibility to overcome longstanding theory-to-praxis problems that teachers face (Bucura and Kruse-Weber, 2021). We challenged group members to consider the necessity of conscious distinction between observation, interpretation and evaluation of video-recorded instrumental/vocal lessons (Rosenberg, 2016). While collaboratively reflecting videos, perception and language were refined. Participants’ perspectives were also taken into account about ambiguity, complexity and diversity in interpretation of their digital content (Bucura and Kruse-Weber, 2021).

We recognize that feedback is a powerful influence on learning and achievement, whether positive or negative (Hattie and Timperley, 2007). Wilkens and Shin (2010) state that peer feedback promotes dialog, encouraging (learners) to see others’ teaching and learning approaches. This can help verbalize one’s own experiences and identify unconscious knowledge, to question and reflect on behaviors and attitudes in music lessons, and to critically examine alternative or innovative ways of thinking and acting. Further considerations for the implementation of ethical principles may emerge with the use of intentional processes, such as a community of practice (Lave and Wenger, 1991). It is a collaborative endeavor, upholding group norms, which ideally facilitates the presence and expression of diverse perspectives as the group works together toward a particular goal.

Further studies should investigate the long-term implications from the workshops. What did the participants change, add, adapt or develop in their teaching and learning personally and as an institution? How did they continue to question their attitudes, values and behavior? Researchers should investigate the extent to which CPD might be compulsory or voluntary and whether it might be effective to include all colleagues in professional development programs or simply individual teachers. In line with Lipowsky and Rzejak (2015) we assume that teachers’ voluntary participation initially might be “more motivated and satisfied. However, there is (still) no evidence that optional participation leads to greater change in teachers’ professional knowledge or instructional quality” (p. 42).

According to Westerlund et al. (2019), we propose being proactive in developing coherent visions for CPD and music education in a sense of societal responsibility, which ought to be created by the music schools themselves, rather than imposed top-down by policy makers and politicians (Westerlund et al., 2019). Music schools should provide “experimental spaces,” and time and facilitation for processes of CPD such as this one. In experimental spaces music schools ought to explore and develop new practices in instrumental/vocal teaching and learning music, “in fulfilling their societal responsibility and promise as game changers” (Gaunt and Westerlund, 2022, xiii).

## Data availability statement

The original contributions presented in the study are included in the article/Supplementary material, further inquiries can be directed to the corresponding author.

## Ethics statement

The requirement of ethical approval was waived by Ethics in Research Advisory Committee at the University of Music and Performing Arts (KUG) for the studies involving humans because after reviewing the projects goals, people, and processes involved, and verifying with the first author, the advisory ethics committee determined that an official review was not necessary for the following reasons: there were no people under the age of 18 involved; all participants freely signed the approved Consent to the Experiment form explaining the use and protection of personal data, and the right withdraw; all consent forms and personal data are stored in a password-protected database accessible only to designated people working on the project. The studies were conducted in accordance with the local legislation and institutional requirements. The participants provided their written informed consent to participate in this study.

## Author contributions

All authors listed have made a substantial, direct, and intellectual contribution to the work and approved it for publication.

## Funding

The project was funded by the Transfer of Knowledge Centre South, respectively, the Austria Wirtschaftsservice with funds from the Nationalstiftung für Forschung, Technologie und Entwicklung (Österreich-Fonds).

## Conflict of interest

The authors declare that the research was conducted in the absence of any commercial or financial relationships that could be construed as a potential conflict of interest.

## Publisher’s note

All claims expressed in this article are solely those of the authors and do not necessarily represent those of their affiliated organizations, or those of the publisher, the editors and the reviewers. Any product that may be evaluated in this article, or claim that may be made by its manufacturer, is not guaranteed or endorsed by the publisher.

## Footnotes

^1^This project in 2016–2018 was funded by the Austrian Federal Ministry for Education, Science and Research and the Austria Wirtschaftsservice.

^2^The project was embedded in a cooperation project involving several Austrian universities called *Connecting.Ideas4Research—participative, inter- and trans-disciplinary knowledge transfer processes between Research and Communities of Practice* (Kleinberger-Pierer et al., 2022).

^3^The term emphasizes that the project started to move outwards—from University to Music Schools. IGP is the German short cut for instrumental- and vocal Leerzeichen statt Bindestrich pedagogy.

^4^The conferences *Challenge Accepted 1.0–4.0* were organized by the team from instrumental and vocal pedagogy at the University of Music and Performing Arts Graz, Austria in 2016, 2017, 2018, 2021.

^5^For the Austrian Music School System (see Lugitsch, 2021, chapter 4.2.1; Rehorska, 2018).

## Abbreviations

CPD: Collaborative professional development
CoP: Community of Practice
CR: Critical Realism
CRP: Critical Response Process
DBR: Design-based research approach
SDT: Self-determination theory
