# Supplementary figures and images for: Facilitating collaborative professional development among instrumental and vocal teachers: A qualitative study with an Austrian Music School
Source: Front Psychol. 2023 Aug 11;13:1096188. doi: 10.3389/fpsyg.2022.1096188 (PMC10457005; doi:10.3389/fpsyg.2022.1096188)

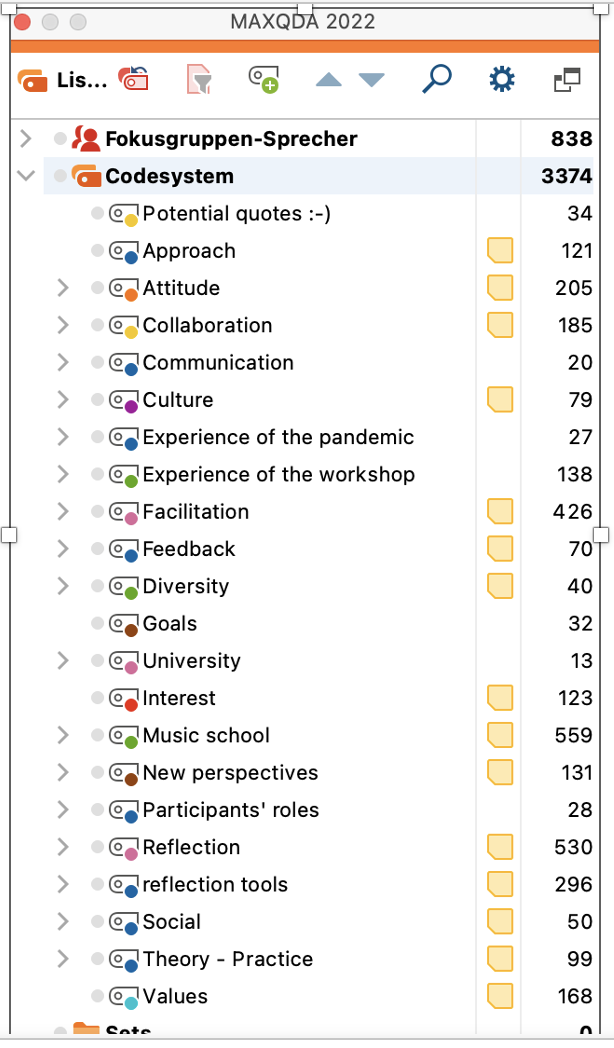

Supplement: Supplementary file 1 [file Image_1.tiff]

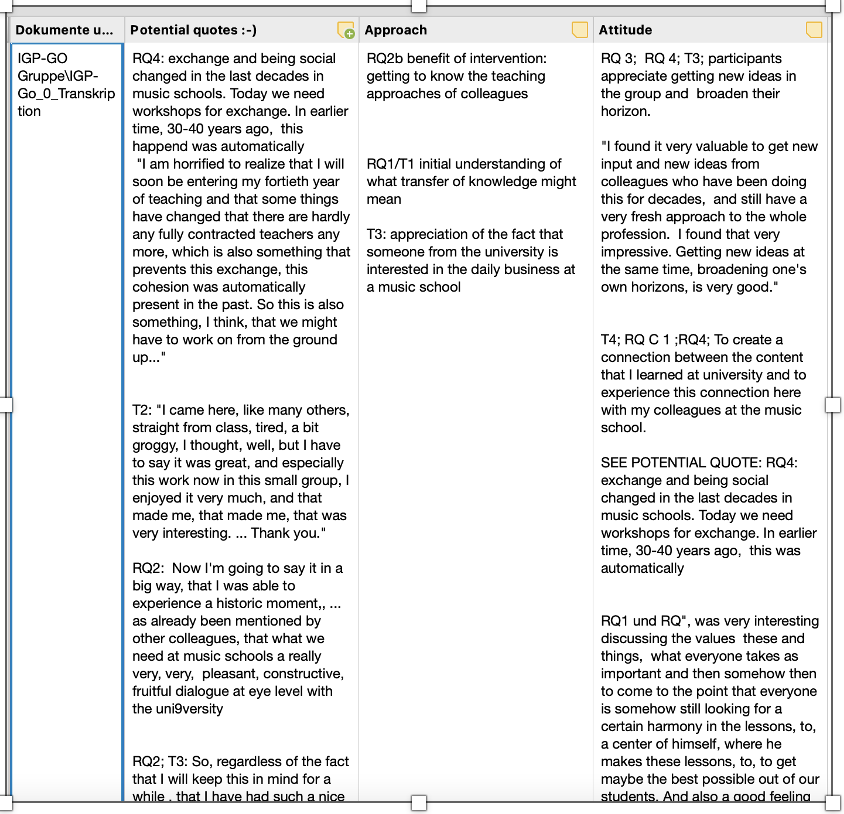

Supplement: Supplementary file 2 [file Image_2.tiff]
